# Comparison of different approaches to small saphenous vein reflux treatment: a retrospective study in two centers

**DOI:** 10.1590/1516-3180.2019.0230.R1.06112019

**Published:** 2020-06-01

**Authors:** Emre Kubat, Celal Selçuk Ünal, Onur Geldi, Erdem Çetin, Aydin Keskin, Kasım Karapınar

**Affiliations:** I MD. Attending Physician, Department of Cardiovascular Surgery, Karabük Training and Research Hospital, Karabük, Turkey.; II MD. Assistant Professor, Department of Cardiovascular Surgery, Karabük Training and Research Hospital, Karabük, Turkey.; III MD. Attending Physician, Department of Cardiovascular Surgery, Zonguldak Atatürk State Hospital, Zonguldak, Turkey.; IV MD. Assistant Professor, Department of Cardiovascular Surgery, Karabük Training and Research Hospital, Karabük, Turkey.; V MD. Attending Physician, Department of Cardiovascular Surgery, Karabük Training and Research Hospital, Karabük, Turkey.; VI MD. Professor, Department of Cardiovascular Surgery, Karabük Training and Research Hospital, Karabük, Turkey.

**Keywords:** Saphenous vein, Laser therapy, Cyanoacrylates, Radiofrequency ablation, Small saphenous vein, Endovenous laser ablation, Stripping

## Abstract

**BACKGROUND:**

Diagnosis and treatment of small saphenous vein (SSV) insufficiency is of utmost importance for relieving chronic venous insufficiency symptoms.

**OBJECTIVES:**

To investigate the efficacy and safety of five different treatment approaches among patients with SSV insufficiency.

**DESIGN AND SETTING:**

Two-center retrospective clinical study, conducted at cardiovascular surgery clinics in a local training and research hospital and a state hospital.

METHODS: A total of 282 extremities of 268 patients with SSV insufficiency alone who were treated for symptomatic varicose veins between January 2012 and January 2017 were included in the study. All extremities included in the study were divided into five groups as follows: high ligation + stripping; radiofrequency ablation (RFA); cyanoacrylate closure (CAC); and endovenous laser ablation (EVLA) at the wavelengths 980 nm and 1,470 nm.

**RESULTS:**

Although the recurrence rate at six months was similar among the treatment groups, we found significant differences in recurrence rates at one year, with lower rates in the CAC, RFA and 1,470 nm EVLA groups, compared with the other treatments (P = 0.005). No sural neuritis was observed in the CAC group. The pigmentation rate was higher in the two EVLA groups (980 nm and 1,470 nm).

**CONCLUSIONS:**

Our study results showed that although CAC, RFA and EVLA at 1,470 nm seemed to be effective methods for treating SSV insufficiency alone, CAC and RFA had better aesthetic results than EVLA at 1,470 nm. We consider that endovenous non-thermal techniques for treating SSV insufficiency may be preferable because of relatively low risk of nerve injury.

## INTRODUCTION

The small saphenous vein has been less suspected in the etiology of venous insufficiency than the great saphenous vein, since it is located in the posterior aspect of the leg with a relatively short length and diameter and less reflux. The prevalence of small saphenous vein insufficiency alone has been found to be 3.5%.^1^ About 20% of patients with venous insufficiency symptoms are diagnosed with small saphenous vein insufficiency.^2^ In particular, a small saphenous vein diameter of ≥ 4 mm has been shown to be associated with venous reflux.^2^ The main symptoms of small saphenous vein insufficiency include pain and burning sensation, itching, heaviness, cramps and restless legs. Symptom severity is closely associated with the degree of chronic venous insufficiency.^3^ Therefore, diagnosis and treatment of small saphenous vein insufficiency is of utmost importance for relieving the symptoms.

The saphenopopliteal junction is located 2 to 4 cm proximally to the popliteal skin crease, where it is included in the popliteal vein, and it is seen in about 83% of the cases. This junction terminates in a normal fashion in only 62% of the cases, since the medial gastrocnemial vessels and small saphenous vein terminate in a common trunk in one-fourth of patients.^4^ In addition, the small saphenous vein is closely connected to the sural nerve from the apex of the calf to the ankle.^4^ Because of this close connection with the sural nerve and a high number of anatomical variations in the popliteal fossa, surgical treatment of small saphenous vein insufficiency is more complicated than is treatment of great saphenous vein insufficiency.^5^

Although the basic surgery for treating small saphenous vein insufficiency consists of ligation and/or stripping, inappropriate or improper ligation results in failure in 22% of the cases with one-year and three-year recurrence rates of 31.6% and 51.7%, respectively.^6^^,^^7^ Over the last decade, endovascular treatment methods have become popular and have been included in the European guidelines for treatment of small saphenous vein insufficiency.^3^ However, no consensus regarding the surgical treatment of small saphenous vein insufficiency has yet been established. Moreover, although the efficacy and safety of different treatments for small saphenous vein insufficiency have already been studied, the number of studies is relatively low, compared with those on great saphenous vein insufficiency. Also, the majority of these studies were limited to head-to-head study designs.

## OBJECTIVE

In the present study, we aimed to investigate the efficacy and safety of five different treatment approaches among patients with small saphenous vein insufficiency alone.

## METHODS

This retrospective study was conducted at cardiovascular surgery clinics in a local training and research hospital and a state hospital between January 2012 and January 2017. A total of 282 extremities of 268 patients who were diagnosed with small saphenous vein insufficiency and underwent conventional surgery or endovenous therapy for symptomatic varicose veins were included.

The inclusion criteria were as follows: ≥ 18 years of age; a small saphenous vein diameter of ≥ 4 mm; saphenopopliteal junction insufficiency grade ≥ 2; Comprehensive Classification System for Chronic Venous Disorders (CEAP) class ≥ 2 and ≤ 5; and complete follow-up data available at six months and one year postoperatively. The exclusion criteria were as follows: a small saphenous vein diameter of < 4 mm; saphenopopliteal junction insufficiency grade < 2; CEAP class < 2 and > 5; and ligation of the small saphenous vein performed alone.

The study protocol was approved by the local ethics committee (date: August 9, 2017; no. 7/11). The study was conducted in accordance with the principles of the Declaration of Helsinki.

All the extremities included in the study were divided into five groups as follows: high ligation + small saphenous vein stripping (n = 45); endovenous laser ablation at the wavelength 980 nm (n = 39); endovenous laser ablation at the wavelength 1,470 nm (n = 36); radiofrequency ablation (n = 134); and cyanoacrylate closure (n = 28) (Table 1).

**Table 1.** Preoperative patient characteristics of the treatment groups

	HLS		980 nm EVLA		1470 nm EVLA		RFA		CAC		
	n	%	n	%	n	%	n	%	n	%	P
Gender											0.101^a^
Female	17	38.6	16	41.0	15	41.7	66	54.5	18	64.3	
Male	27	61.4	23	59.0	21	58.3	55	45.5	10	35.7	
BMI (kg/m^2^)											0.07^a^
< 30	38	86.4	33	84.6	33	91.7	88	72.7	22	78.6	
≥ 30	6	13.6	6	15.4	3	8.3	33	27.3	6	21.4	
CEAP classification											0.2^a^
2	23	52.3	26	66.7	24	66.7	57	47.1	15	53.6	
3	13	29.5	11	28.2	5	13.9	37	30.6	8	28.6	
≥ 4	8	18.2	2	5.1	7	19.4	27	22.3	5	17.9	
Deep venous insufficiency											0.279^a^
No	31	70.5	26	66.7	31	86.1	90	74.4	23	82.1	
Yes	13	29.5	13	33.3	5	13.9	31	25.6	5	17.9	
	n	Mean ± SD (Min-Max) (median)		Mean ± SD (Min-Max) (median)		Mean ± SD (Min-Max) (median)		Mean ± SD (Min-Max) (median)		Mean ± SD (Min-Max) (median)	
Age (year)	44	44.98 ± 10.88 (26-71) (44)	39	44.54 ± 13.62 (26-79) (42)	36	44 ± 12.97 (19-73) (41)	121	45.79 ± 12.16 (19-74) (46)	28	42.96 ±14.04 (18-69) (39)	0.827^b^
SSV diameter (mm)	45	7.07 ± 1.99 (4-13) (6.5)	39	6.5 ± 1.68 (4-11) (6)	36	6.98 ± 1.97 (4-14.2) (6.95)	134	6.65 ± 2.13 (4-13) (6)	28	5.83 ± 1.44 (4-9) (5.4)	0.092^b^

HLS = high ligation + stripping; EVLA = endovenous laser ablation; RFA = radiofrequency ablation; CAC = cyanoacrylate closure, BMI = body mass index; CEAP = comprehensive classification system for chronic venous disorders; SD = standard deviation; Min-Max = minimum-maximum; SSV = small saphenous vein.

^a^chi-square test; ^b^analysis of variance.

The preoperative small saphenous vein diameter, CEAP class, body mass index (BMI), previous history of great saphenous vein surgery, presence of deep venous insufficiency and preoperative and postoperative venous clinical severity scores were recorded. Postoperative procedure-related complications such as bruising, sural neuropathy, thrombophlebitis, pigmentation, skin burns, deep vein thrombosis or pulmonary thromboembolism were also evaluated, along with the severity of postoperative pain and recurrence of venous insufficiency at six months and one year. Data relating to the patients were obtained from the hospital automated record system and from patient files.

The degree of preoperative venous insufficiency was evaluated by the vascular surgeon in accordance with the CEAP classification and venous clinical severity score. The duration of small saphenous vein insufficiency and the vein diameter were assessed using Doppler ultrasonography. Pathological venous reflux was defined as a reverse flow for 0.5 seconds in response to release of calf or thigh compression, with the patient standing, and after a Valsalva maneuver in the supine position.

Preoperative and postoperative (at one year) venous clinical severity scores were calculated for each patient and recorded in the automated system. The severity of postoperative pain was evaluated using a numerical rating scale, i.e. a segmented numerical version of a visual analogue scale, which was found as standard in all patient files.^8^

Sural neuropathy was diagnosed by a neurologist based on clinical examination and electrodiagnostic test results, in patients with typical subjective sensory symptoms such as burning pain, hypesthesia, dysesthesia or paresthesia over the foot or upper calf. Pigmentation was defined as skin color changes during the postoperative period, while persistent pigmentation was defined as the presence of skin pigmentation six months after the operation.^9^

The primary outcome was recurrent varicose veins in treated patients. The postoperative follow-up consisted of clinical examination and Doppler ultrasonography. Recurrence was defined as new-onset varicose veins, subsequent to the procedure.^3^ Success in the procedure was defined as the absence of distal small saphenous vein reflux and absence of neovascularization in the saphenopopliteal junction, as shown using Doppler ultrasonography.

### Intervention techniques

All the operations were performed under spinal anesthesia in the prone position, except for cyanoacrylate closure, which was performed under local anesthesia. Preoperatively, the location of the saphenopopliteal junction was marked on the skin using color Doppler ultrasonography and the patient was then transferred to the operating room. All patients were placed in the prone position.

The standard conventional technique consisted of high ligation + small saphenous vein stripping, which was carried out as previously described by Hong et al.^8^ The endovascular treatment techniques consisted of endovenous laser ablation at the wavelengths 980 nm and 1,470 nm (endovenous laser ablations, FG Group, Ankara, Turkey); radiofrequency ablation (ClosureFast™, Medtronic, USA); and cyanoacrylate closure (VariClose® FG Group, Ankara, Turkey). All of the endovascular catheters were placed from apex of the calf to 2-3 cm distally from the saphenopopliteal junction.

In all thermal techniques, 250-500 ml of tumescent anesthesia (500 ml of normal saline, 15 ml of 2% lidocaine, 20 ml of 8.4% sodium bicarbonate and 0.5 ml of epinephrine [1:1000]) was administered around the small saphenous vein using a 21-gauge needle. The ablation procedure was performed under the guidance of Doppler ultrasonography with application of a local cold pack to the skin. The 980 nm and 1470 nm endovenous laser ablation techniques were performed using 79.6 ± 10.7 (60-100) and 68.2 ± 9.7 (50-90) J/cm, respectively, of laser energy to the vein wall, depending on the diameter of the vein treated.

In the non-thermal procedure, the junction was compressed and collapsed under the guidance of Doppler ultrasonography and glue was continuously injected using a cyanoacrylate system, along the course of the small saphenous vein. Meanwhile, external compression was applied. Once the procedure had been terminated, compression of the small saphenous vein was maintained for an additional 30 seconds. The success rates from the endovascular techniques were evaluated through color Doppler ultrasonography.

### Postoperative follow-up

For all patients who underwent conventional surgery or thermal endovascular treatment, an elastic bandage was used. After the elastic bandage was removed, class 2 (25 to 30 mmHg) compression stockings were applied for six weeks, in accordance with the guideline recommendations.^4^ On the other hand, neither elastic bandages nor compression stockings were applied to the patients who received cyanoacrylate closure.

The severity of pain was evaluated using a numerical rating scale at six hours postoperatively for the patients who were treated under local anesthesia; or at 24 hours postoperatively for the patients who were treated under spinal anesthesia.

During the postoperative period, a single dose of 0.35 ml of tinzaparin sodium (INNOHEP®, Abdi İbrahim, Istanbul, Turkey) was given for prophylaxis of thromboembolism. The patients were examined by a specialist physician, but not by the surgeon who had performed the treatment, in the outpatient clinic at six and 12 months after the operation.

### Statistical analysis

The statistical analysis was performed using the Statistical Package for the Social Sciences (SPSS), version 17.0 software (SPSS Inc., Chicago, IL, USA). Descriptive data were expressed as the mean ± standard deviation (SD) for continuous variables and as numbers and percentages for categorical variables. The chi-square test or Fisher’s exact test was used to compare categorical variables between the groups. Analysis of variance (ANOVA) or the Kruskal-Wallis test was used to assess continuous variables in independent groups, for parametric and nonparametric variables, respectively. A P-value of 0.05 was considered statistically significant.

## RESULTS

A total of 282 extremities of 268 patients who were treated for small saphenous vein insufficiency were included in this analysis. The right lower extremity was treated in 108 patients (40.3%), the left lower extremity in 146 patients (54.3%) and bilateral lower extremities in 14 patients (5.2%).

Among the patients included, 132 (49.3%) were females and 136 (50.7%) were males. The mean age of all the patients was 44.94 ± 12.44 years (range, 18 to 84). The mean body mass index (BMI) was 27.1 ± 3.1 kg/m^2^ (range, 21.2 to 37.3) and 54 patients (20.1%) had a BMI value of ≥ 30 kg/m^2^. The mean diameter of the small saphenous vein of all the patients was 6.65 ± 1.99 cm (range, 4 to 14.2). There was no significant difference among the groups in terms of age, gender, CEAP classification, small saphenous vein diameter, BMI and deep venous insufficiency (Table 1).

Great saphenous vein surgery had previously been performed in 39 patients (14.6%), and there was no significant difference among the groups (P = 0.073). Microphlebectomy was applied to 118 patients (44%), and the number of patients who underwent microphlebectomy was significantly lower in the cyanoacrylate closure group (P < 0.001).

Although there was no significant difference in the recurrence rate at six months among the treatment groups (P = 0.319), we found a statistically significant difference in the recurrence rate at one year. This indicated lower recurrence rates in the cyanoacrylate closure, endovenous laser ablation at the wavelength 1470 nm and radiofrequency ablation groups, compared with the other treatments (P = 0.005) (Table 2).

**Table 2.** Pain scores and recurrence rates of the treatment groups

	HLS		980 nm EVLA		1470 nm EVLA		RFA		CAC		P
	n	Mean ± SD (Min-Max) (median)	n	Mean ± SD (Min-Max) (median)	n	Mean ± SD (Min-Max) (median)	n	Mean ± SD (Min-Max) (median)	n	Mean± SD (Min-Max) (median)	
Pain score (NRS)	44	4.4 ± 1.4 (2-8)	39	3.6 ± 1.9 (0-8)	36	1.9 ± 1.4 (0-6)	121	1.8 ± 1.7 (0-8)	28	0.8 ± 0.9 (0-8)	< 0.001^a^
Recurrence	n = 45	%	n = 39	%	n = 39	%	n = 134	%	n = 28	%	P
6^th^ month	5	11.4	3	7.7	2	5.6	4	3.3	2	7.1	0.319^b^
1^st^ year	14	31.1	9	23.1	4	11.1	13	9.7	3	10.7	0.005^b^

HLS = high ligation + stripping; EVLA = endovenous laser ablation; RFA = radiofrequency ablation; CAC = cyanoacrylate closure; SD = standard deviation; Min-Max = minimum-maximum; NRS = numerical rating scale.

^a^Kruskal-Wallis test; ^b^chi-square test.

In addition, there were statistically significant differences in the numerical rating scale scores among the treatment groups. The pain scores were lowest in the cyanoacrylate closure group (P < 0.001). The numerical rating scale scores were similar in the radiofrequency ablation and 1,470 nm endovenous laser ablation groups, with significantly lower scores than among the patients treated with high ligation + small saphenous vein stripping and with endovenous laser ablation at the wavelength 980 nm (Table 2).

None of the patients experienced major complications. Among all the patients, 33 (11.7%) developed transient sural neuropathy. Two of these patients (n = 1 in the 980 nm endovenous laser ablation group; and n = 1 in the radiofrequency ablation group) had permanent sensory loss along the path of the sural nerve at the end of six weeks, while all neurological symptoms resolved in the remaining patients. Sural neuropathy was most commonly seen in the patients treated with endovenous laser ablation at the wavelength 980 nm (n = 10; 25.6%). None of the patients in the cyanoacrylate closure group had sural neuropathy (Table 3).

**Table 3.** Complications in the treatment groups

Complications	HLS (n = 45)		980 nm EVLA (n = 39)		1470 nm EVLA (n = 36)		RFA (n = 134)		CAC (n = 28)	
	n	%	n	%	n	%	n	%	n	%
Minor complications										
Ecchymosis	8	17.8	1	2.6	0	0	0	0	0	0
Thrombophlebitis	0	0	0	0	0	0	0	0	2	7.1
Pigmentation	0	0	7	17.9	3	8.3	2	1.5	0	0
Sural neuropathy	6	13.5	10	25.6	5	13.9	12	9	0	0

HLS = high ligation + stripping; EVLA = endovenous laser ablation; RFA = radiofrequency ablation; CAC = cyanoacrylate closure.

The rate of ecchymosis was highest in the open surgery group (n = 8; 17.8%). All ecchymoses disappeared by the end of the second week after the operation in all the study groups. Two patients (7.1%) had thrombophlebitis in the cyanoacrylate closure group.

The rate of pigmentation was higher in the endovenous laser ablation groups at the wavelengths 980 nm and 1,470 nm (17.9% and 8.3%, respectively) (Table 3). In the radiofrequency ablation group, there were only two patients (1.5%) with pigmentation. Four patients (10.3%) in the 980 nm endovenous laser ablation group, three patients (7.7%) in the 1,470 nm endovenous laser ablation group and two patients (1.55%) in the radiofrequency ablation group had persistent pigmentation.

There was no significant difference in the preoperative venous clinical severity score scores among the groups (P = 0.493). In addition, there was no significant difference in the postoperative (at one year) venous clinical severity score scores among the groups except for high ligation + small saphenous vein stripping group (P = 0.025). Using each treatment approach, we found a statistically significant clinical improvement in the venous clinical severity score scores, irrespective of the recurrence rate (Table 4).

**Table 4.** Clinical assessment of the treatment groups

	HLS	980 NM EVLA	1470 NM EVLA	RFA	CAC	P
Pretreatment VCSS	4.8 ± 1.4 (2-8)	4.3 ± 1.1 (2-7)	4.5 ± 1.3 (3-8)	4.6 ± 1.4 (2-9)	4.7 ± 1.3 (2-7)	0.493^a^
Post-treatment VCSS	2.2 ± 1.6 (0-5)	1.8 ± 1.3 (0-5)	1.5 ± 1.3 (0-4)	1.4 ± 1.4 (0-7)	1.4 ± 1.2 (0-4)	0.025^a^
P	< 0.001^b^	< 0.001^b^	< 0.001^b^	< 0.001^b^	< 0.001^b^	

VCSS = venous clinical severity score; EVLA = endovenous laser ablation; RFA = radiofrequency ablation; CAC = cyanoacrylate closure.

^a^Kruskal-Wallis test; ^b^Wilcoxon signed-rank test.

## DISCUSSION

Over recent years, endovenous techniques have become popular and have been increasingly used for treating small saphenous vein insufficiency, as an alternative to surgery. Because of the anatomical variations of small saphenous vein and the difficult nature of this surgery, the success rate from conventional surgery is relatively low.^6^^,^^7^ With increasing use of endothermal ablation techniques and with increasing familiarity with Doppler ultrasonography among vascular surgeons in daily practice, the success rates of the procedure have increased, compared with conventional surgery, for patients with small saphenous vein insufficiency.

In a previous study, the recurrence rates were reported to be 31.6% and 51.7% at one and three years, respectively, among patients undergoing small saphenous vein ligation and/or stripping.^7^ In a recent study, however, the recurrence rate was shown to be 4.3% among patients undergoing modified high ligation and segmental stripping, although the sample size was small.^10^ In our study, on the other hand, the recurrence rate was 31.1% among patients undergoing high ligation + segmental stripping. This can be attributed to the small sample size and to the fact that endovascular treatment methods were not popular at the beginning of the endovascular era when most patients underwent conventional surgery. Moreover, the small saphenous vein anatomy could not be evaluated accurately, given that vascular surgeons were only rarely familiar with intraoperative Doppler ultrasonography guidance.

Today, endovenous laser ablation and radiofrequency ablation are the most common endothermal ablation techniques. In one study, the obliteration rate of radiofrequency ablation was found to be 93.4%, one year after the procedure.^11^ In the literature, there are several studies reporting success rates of 100%, one year after the procedure, among patients treated with endovenous laser ablation at the wavelength 1,470 nm.^12^^,^^13^ In a systematic meta-analysis that included 49 studies on patients with small saphenous vein insufficiency, the anatomical success rate was 58.0% among 798 patients treated with surgery, 98.5% among 2,950 patients treated with endovenous laser ablation and 97.1% among 386 patients treated with radiofrequency ablation.^14^ Most recently, cyanoacrylate closure has been introduced for treating venous disorders. In a meta-analysis on previous studies, the success rate was found to be 96.8%, one year after the procedure, among 53 patients with small saphenous vein insufficiency.^15^ In our study, the success rates from the procedures of radiofrequency ablation, cyanoacrylate closure and endovenous laser ablation at the wavelength 1,470 nm (90.3%, 89.3% and 88.9%, respectively) were found to be higher than the rates for the other two treatment groups, one year after the procedure. Our success rate from radiofrequency ablation was consistent with data in the literature, but our rates relating to cyanoacrylate closure and endovenous laser ablation at the wavelength 1,470 nm were somewhat lower than those from previous studies. We consider that the smaller sample size of the groups that underwent cyanoacrylate closure and endovenous laser ablation at the wavelength 1,470 nm, compared with the size of the radiofrequency ablation group, may have affected our results relating to anatomical success rate.

Age, gender and obesity (particularly BMI > 30 kg/m^2^) are well-known risk factors for venous insufficiency.^3^ In addition, risk factors such as the diameter of the treated vein, deep venous insufficiency, preoperative CEAP classification and type of device used have been shown to be associated with treatment failure from endovascular treatment methods for venous insufficiency.^3^^,^^16^^,^^17^ Casana et al.^18^ showed that postoperative vein reduction after the radiofrequency ablation procedure was influenced by preoperative CEAP class. In our study, there was no significant difference among the groups in terms of age, gender, CEAP classification, small saphenous vein diameter, BMI or deep venous insufficiency.

In one study, the success rate from the procedure was reported to be 97% at six weeks, among patients with small saphenous vein insufficiency that was treated with laser ablation at the wavelength 980 nm. However, in that study, it was only possible to evaluate 60% of the patients.^19^ In another study, Park et al. showed that the success rate from the procedure was 94%, one year after the procedure, among patients with small saphenous vein insufficiency that was treated with endovenous laser ablation at the wavelength 980 nm, although it was only possible to evaluate 40% of the patients.^20^ In addition, previous studies revealed that the anatomical success rate was similar between the endovenous laser ablation procedures at the wavelengths 980 nm and 1,470 nm.^14^^,^^21^ In contrast, we found a significant lower success rate with endovenous laser ablation at 980 nm, compared with the laser at 1,470 nm. In our study, the success rate from the endovenous laser ablation procedure at the wavelength 980 nm was found to be 76.9% at one year, i.e. a lower rate than previous findings. This can be explained by the fact that our sample size was relatively small and nearly half of the patients undergoing endovenous laser ablation at the wavelength 980 nm in studies in the literature could not be evaluated.^19^^,^^20^

Postoperative procedure-related complications reported previously have included bruising, sural neuropathy, thrombophlebitis, pigmentation, ecchymosis, skin burns, deep vein thrombosis or pulmonary thromboembolism, among patients with small saphenous vein insufficiency.^3^ In our study, no major complications such as skin burns, deep vein thrombosis or pulmonary embolism were seen in any of the patients. Pigmentation resulted in poor esthetic results and reduced the quality-of-life scores of the treated patients.^3^

Along the natural course of the small saphenous vein, it runs just below the skin and shows a wide range of variations. Therefore, thermal injury-related skin changes are more common. Although hyperpigmentation was reported in 5% of the patients after the endovenous laser ablation procedure, hyperpigmentation along the ablated vein after the endovenous laser ablation procedure occurs in up to 12% of the patients.^3^^,^^22^ On the other hand, hyperpigmentation was reported in 3 to 4% of the patients in the literature, after radiofrequency ablation.^3^^,^^18^ However, these results were usually given as great saphenous vein treatment results. The hyperpigmentation rate was reported to be 3.3% after endovenous laser ablation surgery to treat small saphenous vein reflux in one randomized clinical trial.^23^ The pigmentation rate was higher in the two endovenous laser ablation groups (wavelengths 980 nm and 1,470 nm) in our study (17.9% and 8.3%, respectively). A total of nine patients (n = 4 with 980 nm endovenous laser ablation; n = 3 with 1,470 nm endovenous laser ablation; and n = 2 with radiofrequency ablation) presented persistent pigmentation. These results were higher than what has been reported in the literature and can be attributed to the fact that pigmentation rates were usually not mentioned in previous studies on small saphenous vein surgery. Skin complication rates seem to be higher with endovascular thermal treatment of small saphenous vein insufficiency.

Ecchymosis is an early postoperative complication that is associated with poor pain and quality-of-life scores.^3^ In the literature, the rate of ecchymosis has been reported as 3% to 4%, among patients undergoing endovascular treatment of small saphenous vein insufficiency.^3^ In our study, the patients who underwent high ligation + small saphenous vein stripping had the highest ecchymosis rate (17.85%), while only one patient (2.6%) in the group with endovenous laser ablation at the wavelength 980 nm developed ecchymosis. The higher rate of ecchymosis in the high ligation + small saphenous vein stripping group can be explained by the high number and variety of small saphenous vein deep venous vascular connections. Because the small saphenous vein is adjacent to the sural nerve, sural nerve injury can be seen during treatment. Sural nerve injury is associated with a burning sensation in the innervation site, numbness and sensory loss, leading to neuroma formation.^3^ Although previous studies have demonstrated divergent results, it was found through a meta-analysis that the rates of sural nerve injury from conventional surgery, endovenous laser ablation and radiofrequency ablation were 19.6%, 4.8% and 9.7%, among patients with small saphenous vein insufficiency.^14^

In this context, it is of utmost importance to identify the entry site of the affected saphenous vein. The small saphenous vein is closest to the sural nerve under the mid-calf. In one study, access from the lateral malleolus rather than from the calf was found to be associated with a higher rate of paresthesia, among patients treated with endovenous thermal ablation.^24^ In contrast, Sanioglu et al. reported that access from the mid-calf was not safe and suggested that the nerve should be identified under the guidance of ultrasonography.^25^ In our study, the rate of neurological sequelae was highest among the patients treated with endovenous laser ablation at the wavelength 980 nm. The rate of neurological sequelae was similar between the other treatment groups, except in the cyanoacrylate closure group, in which no sequelae were seen. The higher rate of neurological complications can be attributed to the fact that endovenous laser ablation at the wavelength 980 nm was previously the most frequently applied treatment within clinical practice.

Our results regarding sural nerve injury seem to be consistent with the findings in the literature regarding endovascular thermal treatment and conventional surgery. Of note, we believe that experience is more important than the access site, in using endothermal techniques, for minimizing neurological complications. In addition, the severity of pain was evaluated on a numerical rating scale among the treatment groups, and the cyanoacrylate closure group presented the lowest pain scores. The radiofrequency ablation and endovenous laser ablation at 1,470 nm showed similar scores. The lack of microphlebectomy in the cyanoacrylate closure group might have contributed to lower pain scores. According to our study results, although all the treatment techniques were performed under spinal anesthesia except for cyanoacrylate closure, the radiofrequency ablation, endovenous laser ablation at 1,470 nm and cyanoacrylate closure techniques seemed to be associated with less pain.

In our study, the venous clinical severity score was used to assess per-operative clinical improvement. We found that there was a statistically significant improvement in the venous clinical severity score scores postoperatively in all procedures, irrespective of the recurrence rate. Although the high ligation + small saphenous vein stripping group showed a statistically significant postoperative clinical improvement, this group had the highest postoperative venous clinical severity score among the treatment groups, because of the high recurrence rate.

There were some limitations to the present study. It had a small sample size with unequal sizes among the treatment groups. In addition, it was not possible to thoroughly evaluate patient satisfaction due to missing data in the quality-of-life questionnaires. Although the retrospective design can be deemed to be another limitation, we believe that this study will provide additional information for the body of knowledge on this subject, given that no head-to-head studies comparing five different methods for treating small saphenous vein insufficiency alone are available in the literature.

## CONCLUSION

Our study showed that although cyanoacrylate closure, radiofrequency ablation and endovenous laser ablation at the wavelength 1,470 nm seemed to be effective methods for treating small saphenous vein insufficiency alone, cyanoacrylate closure and radiofrequency ablation had better esthetic results than those from endovenous laser ablation at 1,470 nm. Although complication rates tend to decrease with increasing experience in endovascular procedures over time, thermal ablation therapies will always imply a risk of neurological complications. Therefore, we consider that endovenous non-thermal techniques for treating small saphenous vein insufficiency may be preferable because of their relatively low risk of nerve injury.
